# Mechanisms of acetaminophen hepatotoxicity and their translation to the human pathophysiology

**DOI:** 10.18053/jctres.03.2017S1.002

**Published:** 2017-02-12

**Authors:** Anup Ramachandran, Hartmut Jaeschke

**Affiliations:** Department of Pharmacology, Toxicology & Therapeutics, University of Kansas Medical Center, Kansas City, United States

**Keywords:** acetaminophen, hepatotoxicity, protein adducts, mitochondria, oxidative stress, nitric oxide, DNA fragmentation, regulated necrosis, mitochondrial dynamics, biomarkers

## Abstract

Acetaminophen (APAP) overdose is the most common cause of acute liver failure in the United States and mechanisms of liver injury induced by APAP overdose have been the focus of extensive investigation. Studies in the mouse model, which closely reproduces the human condition, have shown that hepatotoxicity is initiated by formation of a reactive metabolite *N*-acetyl-*p*-benzoquinone imine (NAPQI), which depletes cellular glutathione and forms protein adducts on mitochondrial proteins. This leads to mitochondrial oxidative and nitrosative stress, accompanied by activation of c-jun *N*-terminal kinase (JNK) and its translocation to the mitochondria. This then amplifies the mitochondrial oxidant stress, resulting in translocation of Bax and dynamin related protein 1 (Drp1) to the mitochondria, which induces mitochondrial fission, and ultimately induction of the mitochondrial membrane permeability transition (MPT). The induction of MPT triggers release of intermembrane proteins such as apoptosis inducing factor (AIF) and endonuclease G into the cytosol and their translocation to the nucleus, causing nuclear DNA fragmentation and activation of regulated necrosis. Though these cascades of events were primarily identified in the mouse model, studies on human hepatocytes and analysis of circulating biomarkers from patients after APAP overdose, indicate that a number of mechanistic events are identical in mice and humans. Circulating biomarkers also seem to be useful in predicting the course of liver injury after APAP overdose in humans and hold promise for significant clinical use in the near future.

**Relevance for patients**: This review focuses on the mechanisms behind APAP-induced hepatotoxicity and the relevance of these to the human pathophysiology. Current investigations on various biomarkers which may be useful in clinical management of APAP overdose patients are also discussed.

## 1. Introduction

Acetaminophen is an analgesic drug, which is safe at therapeutic doses, but can produce significant hepatotoxicity with an overdose. APAP hepatotoxicity is the most frequent cause of acute liver failure (ALF) in the US [1] and a recent study evaluating outcomes in adults with acute liver failure between 1998 and 2013 indicates that hepatotoxicity due to APAP accounted for almost half the cases of ALF for the entire 16-year period, with unintentional APAP overdoses, (those in which patients took excessive medication over several days for ailments like pain or fever) being more common than intentional (suicidal) overdoses [2]. This is similar to an earlier study, which showed that unintentional APAP overdose accounted for over 50 % of cases of acetaminophen-related ALF [3]. These unintentional overdoses are mainly driven by the increasing availability of combination drugs, which contain acetaminophen in addition to other drug classes such as opioids [4]. Once the hepatotoxicity due to APAP was recognized, the mechanisms behind this were extensively investigated, especially in the mouse model, which recapitulates key mechanistic aspects of liver injury in humans such as mitochondrial dysfunction [5,6]. These insights, especially regarding GSH depletion and protein adduct formation [7], resulted in development of *N*-acetylcysteine (NAC) as an antidote for APAP overdose [8,9]. NAC treatment is now the standard of care under these conditions, and in spite of the significant therapeutic potential of NAC in preventing APAP- induced ALF, it has to be administered early after APAP consumption to have the most benefit. Since this may not be possible for most patients with an APAP overdose, especially those with unintentional overdoses, new therapeutic options which could benefit when administered at later time points are continuously being investigated. These studies have led to significant additional insight into cellular signaling events driving hepatocyte death and liver injury after APAP overdose and will be reviewed in this article.

## 2. Acetaminophen metabolism initiates liver injury.

Therapeutic doses of APAP are typically conjugated with glucuronic acid and sulfate, which are the main metabolites of APAP, and are then excreted in the urine [10]. A minor component of APAP is also oxidized by the microsomal cyto-chrome P450 system, predominantly by Cyp2E1 and Cyp1A2 [11], to form a reactive metabolite, *N*-acetyl-*p*-benzoquinone imine (NAPQI) [12]. This minor metabolite is typically harmless, since it is mostly conjugated with glutathione and excreted in bile [13,14]. However, even at these low, therapeutic doses, there occurs very limited reaction with protein sulfhydryl groups leading to covalent binding and protein adduct formation [15,16]. The impact of these protein adducts is limited because they are effectively removed by autophagy [17]. It is only when high levels of APAP saturate the sulfation pathway and glucuronidation cannot keep up any longer [18], that there is an excess generation of the reactive NAPQI metabolite. This then consumes glutathione for its conjugation, resulting in depletion of glutathione stores. While the initial depletion is similar in both GSH and GSSG, without affecting the GSSG:GSH ratio (1:200), the recovery rates are different, with GSSG content increasing faster than that of GSH [19]. Recovery rate of GSH can significantly influence injury, since an induction of glutamate-cysteine ligase which correlated with faster recovery of GSH is one of the mechanisms by which female mice are protected against APAP-induced liver injury [20]. A differential metabolomics study suggests that the depletion of glutathione after low dose APAP (150mg/kg) is paralleled by elevation in the glutathione analogue ophthalmic acid, where the SH group of the cysteine residue of GSH is replaced with a CH3 group from 2-aminobutyrate [21]. Detection of ophthalmic acid in serum from APAP-induced acute liver failure patients was also more frequent in non-survivors [22]. As glutathione depletion occurs, there is an increasing reaction of NAPQI with sulfhydryl groups of proteins to form protein adducts [15]. In contrast to earlier assumptions, this generation of protein adducts can take place before GSH levels are depleted extensively [15,23] and also after therapeutic doses of APAP without relevant GSH depletion [15,16]. Interaction of NAPQI with mitochondrial proteins and formation of mitochondrial protein adducts is thought to be critical for the toxicity [24-26].

## 3. Mitochondrial protein adduct formation and APAP hepatotoxicity.

Though mitochondria were traditionally considered important cellular organelles due to their role in ATP generation, it is now evident that they play important roles in various cell signaling scenarios, including cell death by regulated necrosis [27]. Formation of NAPQI adducts on mitochondrial proteins was found to be unique to APAP when compared to its regioisomer 3’-hydroxyacetanilide (AMAP), which is non-toxic in mice [24,28,29]. In contrast, recent reports indicate that AMAP is cytotoxic in primary human hepatocytes or precision-cut human liver slices [30,31]. Interestingly, AMAP toxicity in primary human hepatocytes correlated with mitochondrial protein adduct formation and mitochondrial dysfunction [31]. Specific targets within the mitochondria, such as glutathione peroxidase and the alpha subunit of ATP synthase have been identified to undergo adduct formation by proteomic approaches [32]. Enzymes such as HMG CoA synthase have also been shown to be modified, accompanied by inhibition of enzyme activity [33]. APAP adducts on mitochondrial proteins such as glycine amidinotransferase, Parkinson disease protein 7 (PARK7), peroxiredoxin 6 and voltage-dependent anion-selective channel protein 2 (VDAC2) have also been detected in cultures of human hepatocytes [34], indicating that adduct formation is not a global phenomenon affecting all mitochondrial proteins, but rather selective with specific targets. While mitochondrial protein adducts are relevant to APAP hepatotoxicity since NAPQI binding to mitochondrial proteins correlates with the toxicity [35], the role of specific proteins targeted for adduct formation in the pathophysiology is not well understood.

## 4. Adduct formation initiates mitochondrial oxidative and nitrosative stress

**Figure 1.**
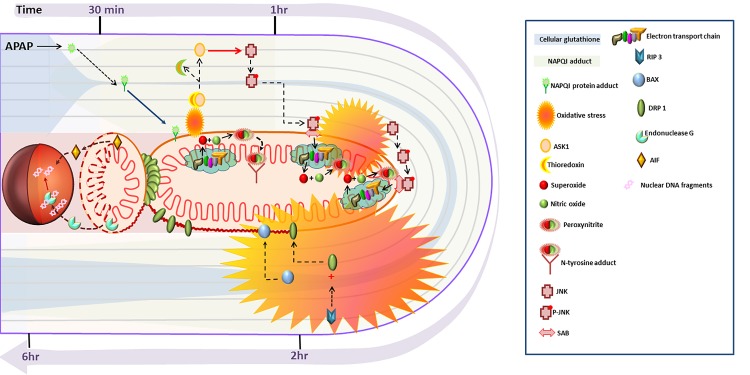
A Minardo representation [167] of acetaminophen hepatotoxicity in the mouse, illustrating the temporal separation of events after an APAP overdose. APAP hepatotoxicity is initiated by its conversion to the reactive intermediate NAPQI, which results in glutathione depletion and formation of APAP protein adducts. Adduct formation on mitochondrial proteins modulates respiratory chain function, producing elevated levels of free radicals such as superoxide. This, along with nitric oxide, generates peroxynitrite resulting in protein nitration within mitochondria. Mitochondrial oxidative stress results in oxidation of thioredoxin 1, releasing its partner ASK1, which activates JNK resulting in its phosphorylation and translocation to the mitochondrial outer membrane, where it interacts with Sab and subsequently stimulates free radical production from the mitochondrial electron transport chain. This in turn amplifies JNK activation and subsequent mitochondrial oxidant stress, which ultimately results in activation of RIP3 and translocation of Drp1 and Bax to the mitochondria. While Bax initiates outer membrane permeabilization, Drp1 induces mitochondrial fission and subsequent activation of the mitochondrial permeability transition. This then releases apoptosis inducing factor (AIF) and endonuclease, which translocate to the nucleus and initiates nuclear DNA fragmentation.

Mitochondrial protein adduct formation results in increased superoxide production [36] (Figure 1) accompanied by compromised mitochondrial respiration [37]. The importance of mitochondrial superoxide production in APAP hepatotoxicity is illustrated by the fact that mice with a partial deficiency of manganese superoxide dismutase (SOD2) (which would usually scavenge superoxide) have exaggerated liver injury when exposed to APAP overdose [38,39]. The enhanced production of superoxide would allow its reaction with nitric oxide (NO) within the mitochondria producing the reactive radical peroxynitrite (ONOO^–^), which can nitrate protein tyrosine residues and compromise their function [40] (Figure 1). APAP hepatotoxicity results in early nitrotyrosine formation exclusively in hepatocyte mitochondria [41], suggesting that generation of elevated superoxide and its reaction with NO occurs within this organelle. The source of nitric oxide contributing to peroxynitrite formation within mitochondria, however, is not well characterized. It has been demonstrated that peroxynitrite formation subsequent to APAP overdose is independent of inducible nitric oxide synthase (iNOS) [42]. The lack of iNOS involvement was also suggested by the absence of protection with iNOS inhibitors [43] and in iNOS-deficient mice [44]. In contrast, neuronal NOS (nNOS) was shown to be present in hepatocytes [45] and a nNOS inhibitor was protective against APAP-induced cell death in isolated mouse hepatocytes [46]; likewise, nNOS-deficient mice showed delayed injury after APAP overdose [47], suggesting that nNOS could be a putative source of NO for peroxynitrite formation after APAP overdose.

The relevance of peroxynitrite to APAP-induced hepatotoxicity is illustrated by the critical anti-oxidant proteins such as SOD2 it targets [48] and the protection afforded by its scavenging with delayed glutathione supplementation, which also replenished mitochondrial GSH levels [49-52]. In addition, the selective metabolism of superoxide by the mitochondrial targeted SOD-mimetic Mito-TEMPO effectively reduced APAP hepatotoxicity [53]. Furthermore, APAP overdose in SOD2 deficient mice also caused aggravated liver injury accompanied by exacerbated peroxynitrite and protein carbonyl formation [38,39].

## 5. The MAP kinase JNK and amplification of mitochondrial oxidative stress.

While formation of APAP-protein adducts on mitochondria initiates a mitochondrial nitrosative and oxidative stress, this effect alone does not seem to be sufficient to ultimately trigger the MPT and cell death. Thus, it was recognized that an oxidant stress-mediated activation of the MAP kinase JNK in the cytosol [54] is what ultimately seems to amplify the mitochondrial oxidant stress and results in downstream signaling events. JNK activation after APAP overdose occurs early after APAP overdose, and is then sustained during the signaling cascade inducing hepatocyte cell death. The apoptosis signal-regulating kinase 1 (ASK1) is involved in APAP-induced activation of JNK, with ASK1- deficient mice being protected against the sustained JNK elevation [55] and a specific ASK1 inhibitor decreasing JNK activation at 1.5 h and preventing JNK translocation to mitochondria [56]. In addition, it was found that the mixed-lineage kinase 3 (MLK3) was activated by oxidative stress and was required for JNK activation in response to oxidative stress [57]. It was also seen that JNK phosphorylation at one, three and six hours after APAP treatment was significantly attenuated in MLK3-KO mice [57]. Since MLK3 has been suggested to be part of a feedback mechanism that regulates cellular responses to ROS [58] and the activation of JNK can be prevented by anti-oxidants [42], it is possible that APAP-induced, ROS mediated JNK activation occurs through multiple mechanisms with temporal changes in their interaction.

Though activation of JNK in the cytosol seems to be an early event after APAP overdose, occurring within an hour after a 300 mg/kg dose in the mouse [56], it is also influenced by the dose of APAP. Lower APAP doses such as 150mg/kg were shown to induce transient JNK activation with reversible mitochondrial dysfunction in the mouse liver [26]. Once JNK is activated and phosphorylated in the cytosol, the next mechanistic step for amplification of mitochondrial injury is its translocation to mitochondria, where it binds to the Sab protein on the outer mitochondrial membrane [54,59]. Binding to and phosphorylation of Sab by p-JNK leads to inactivation of p-Src on the inner mitochondrial membrane, which then inhibits electron transport and increases reactive oxygen species release [54,60], thus amplifying oxidant stress and peroxynitrite formation [42]. While a few studies suggested that JNK was protective in acetaminophen toxicity [61,62], the data from one of them [62] could be influenced by the differing susceptibility to APAP toxicity of mice sub-strains used in the study [63]. It is however unclear why the knocking down of both JNK1 & 2 in hepatocytes resulted in a paradoxical exacerbation of APAP-induced liver injury [61], and this requires further study. The overall pathophysiological relevance of the JNK amplification loop in the murine models of APAP- induced liver injury has been extensively shown with various JNK inhibitors and JNK gene silencing [42,64,65] as well as the protection by inhibition of upstream signaling events [56,57,59]. Similar effects of APAP-induced JNK activation and mitochondrial p-JNK translocation was also observed in primary human hepatocytes although JNK inhibition only moderately reduced cell death [66]. It remains unclear if this is due to a species difference or reflects the greater dependence on oxidant stress amplification *in vivo* compared to cultured cells, which are generally kept under hyperoxic conditions resulting in more oxidant stress [36].

In parallel to activation and translocation of JNK, mitochondrial oxidant stress also results in the early translocation of the cytosolic protein Bax to the mitochondria [67,68]. While the initial mitochondrial oxidant stress seems to be required for Bax translocation [53], Bax does not seem to subsequently influence mitochondrial oxidant stress and peroxynitrite formation, since the protection against APAP-induced liver injury in Bax knockout mice was transient, only occurring at early time points [67]. However, recent information suggesting that Bax could be part of a large integrated network mediating various regulated forms of cell death through mitochondrial translocation [69] give tantalizing clues as to its implications in APAP-induced liver injury and will be further discussed below.

## 6. APAP overdose, mitochondrial dynamics and autophagy.

Mitochondria are dynamic organelles which undergo changes in morphology through cycles of fusion and fission. These processes are critical for mitochondrial homeostasis and bioenergetics [70]. Mitochondrial fusion and fission are regulated by a number of GTPase proteins such as optic atrophy 1 (OPA1), mitofusin 1/2 (Mfn1/2) and dynamin related protein 1 (Drp1) in mammalian cells [71]. While OPA1 and Mfn1 & 2 are involved in mitochondrial fusion, Drp1 mediates mitochondrial fission in mammals, where the role of mitochondrial fission 1 protein (Fis1) (a fission protein in yeast) is still not confirmed [72]. The impact of APAP overdose on mitochondrial dynamics was first identified when significant elevations in Drp1 and its translocation to the mitochondria were discovered after APAP overdose [73] (Figure 1). The role of Drp1 in mitochondrial fission after APAP overdose was subsequently confirmed by other studies [74], suggesting that alterations in mitochondrial dynamics after APAP overdose could have important mechanistic implications. Interactions between Bax and Drp1 have also been implicated during mitochondrial fission in pathophysiological conditions, with spatial and temporal association of Bax with mitochondrial fission sites, Drp1, and Mfn2 during apoptosis [75]. Bax has been suggested to be required for Drp1-mediated mitochondrial fission caused by photodynamic therapy in human lung adenocarcinoma cells [76], while Drp1 influenced Bax translocation to mitochondria in response to irradiation-induced apoptosis [77] and Drp1-induced membrane remodeling stimulates Bax oligomerization [78]. In addition, pharmacological inhibition of Drp1was shown to prevent Bax induced mitochondrial outer membrane permeabilization (MOMP) [79]. This interaction between Bax and Drp1, both of which translocate to the mitochondria after APAP overdose would suggest a scenario where APAP-induced Bax and Drp 1 translocation to mitochondria facilitate mitochondrial fission, which then initiates down-stream events such as opening of the mitochondrial permeability transition pore.

Removal of damaged mitochondria through autophagy (mitophagy) has been shown to limit APAP-induced injury [80], especially adjacent to the acute necrosis area [81]. Mitochondrial fission as observed during APAP hepatotoxicity may enhance this process [73]. The mitochondrial translocation of Parkin, an E3 ubiquitin ligase, is required for mitophagy induction after APAP overdose and acute knockdown of Parkin aggravates APAP-induced liver injury [82]. However, chronic deletion of Parkin renders animals resistant to APAP [82], possibly due to development of compensatory and adaptive mechanisms for the chronic loss of Parkin, which may contribute to the resistance to APAP-induced liver injury [82]. Parkin-independent mitochondrial spheroid formation may substitute for Parkin-dependent autophagy in removing damaged mitochondria [83]. On the other hand, deletion of the autophagy gene Atg5 leads to chronic injury, regeneration and inflammation, which also protected against APAP toxicity [84]. The mechanism of this protection involves the persistent activation of Nrf2 with higher GSH synthesis rates and increased hepatocyte proliferation [84]. These observations support the critical role of autophagy for cell survival under normal conditions and during APAP-induced liver injury.

Protein folding in the endoplasmic reticulum (ER) is a critical cellular function and various cellular stresses such as ROS or alterations in cellular calcium can impair protein folding and initiate ER stress. Mice treated with 200mg/kg of acetaminophen showed activation of ER stress with upregulation of GADD153/CHOP by 6 hours after APAP administration, accompanied by a decrease in Grp78 levels [85]. Higher doses of APAP also induce markers of ER stress, with doses of 450mg/kg APAP inducing activation of ER stress-responsive transcription factor ATF6 and transcriptional activation and elevated expression of GADD153/CHOP [86]. CHOP deficient mice were also shown to be protected against APAP-induced liver injury, though interestingly the protection was only seen in animals given APAP by gavage and not in those given APAP as an intra-peritoneal injection [87]. Hence, while ER stress does seem to occur after APAP overdose, the mechanisms by which APAP induces ER stress are poorly understood [88] and need more study.

## 7. APAP-induced mitochondrial permeability transition and regulated necrosis.

While the immediate consequence of amplification of mitochondrial oxidant stress is loss of mitochondrial protein function due to modification of thiols [33], peroxynitrite mediated nitrotyrosine formation [48] and oxidative mitochondrial DNA damage [41], the critical event resulting in escalation of cell wide damage is induction of the mitochondrial permeability transition (MPT). The mitochondrial permeability transition is an extensively studied phenomenon, which was initially linked to the apoptotic cell death pathway, though it is now recognized that it is activated in numerous forms of cell death. It involves initial permeabilization of the mitochondrial outer membrane, followed by an abrupt change in inner membrane permeabilization allowing exit of molecules less than 1500 daltons [89,90]. Though it has been suggested that Bax and Bak form the components of the mitochondrial permeability transition pore (MPTP) in the outer membrane [90,91], the components within the inner membrane are still being confirmed. While cyclophilin D is generally accepted as being one of the proven components and regulator of the MPT [92], recent evidence suggests that the c-subunit ring of the F1FO ATP synthase could also be a regulatory unit within the inner membrane [93]. The induction of the MPT subsequent to APAP overdose results in breakdown of the proton gradient across the membrane, loss of mitochondrial membrane potential, and consequently, cessation of ATP production, which eventually leads to cell death [94-97]. However, the role of cyclophilin D and the MPTP in APAP-induced liver damage seem to be dependent on the dose of APAP administered. For example, while use of a relatively low overdose of 200 mg/kg APAP demonstrated protection against liver injury in cyclophilin D-deficient mice [96], only transient protection was achieved when cyclophilin was inhibited using cyclosporine A *in vitro* [94]. Furthermore, treatment with a higher dose of APAP (600 mg/kg) *in vivo* also showed no protection against liver injury [98]. Though MPT was initially studied only in the context of pathophysiology, it is now being recognized that it could also have physiological roles [99], such as in the heart [100] and in calcium buffering in neuronal cells [101]. This may explain the transient MPTP opening seen after treatment of mice with low doses of APAP (150mg/kg) [26], when JNK activation was also seen only for a short term. However, sustained JNK activation resulted in irreversible activation of MPT [26]. The involvement of lysosomal iron translocating to the mitochondria through the calcium uniporter has also been suggested in APAP-induced MPTP opening [102,103] implying that reactive oxygen species formation through the iron-mediated Fenton reaction could also be an amplifying source to ultimately cause mitochondrial dysfunction. Hence, the activation of the mitochondrial permeability transition pore, which is a key mechanistic step in the cascade of cell signaling after APAP overdose, seems to be influenced by the dose of APAP, with lower doses producing a reversible activation, while higher doses produce a sustained effect.

The immediate consequence of Bax-induced permeabilization of the mitochondrial outer membrane would be release of a number of proteins from the inter-membrane space, which have been implicated in cell death pathways such as apoptosis. Release of these proteins including cytochrome c, *s*econd *m*itochondria-derived *a*ctivator of *c*aspase (Smac), apoptosis inducing factor (AIF) and endonuclease G have been extensively studied in the context of apoptotic cell death, and it is recognized that the mechanisms of their release may not be identical in all cases. Cytochrome c and Smac release occurs almost simultaneously, suggesting that they exit mitochondria by a similar mechanism [104], while AIF release from the mitochondria occurs at a different rate [105]. Also, while Bax was involved in release of cytochrome c and Smac, this was not the case for endonuclease G and AIF [106], suggesting that these two proteins may be released by slightly differing mechanisms in contrast to cytochrome c and Smac. It has also been shown that DRP1 can influence release of intermembrane proteins [104] implicating it in release of cytochrome c, Smac, AIF and endonuclease G which is evident after APAP [67,107]. Once in the cytosol, AIF and endonuclease G (both of which contain nuclear localization sequences [108]) translocate to the nucleus [107], where endonuclease G cleaves DNA every 50-300kb [109], thereby generating DNA fragments typically seen after APAP overdose [41,110] (Figure 1). AIF is also critical for DNA damage and liver injury due to its involvement in DNA fragmentation [111], a fact highlighted by the fact that AIF-deficient mice have lower DNA damage and liver injury after APAP [112].

## 8. Necrotic cell death after APAP overdose

As discussed, proteins released from the mitochondrial inter-membrane space after APAP are also seen in the cytosol after apoptotic cell death. However, despite the mitochondrial release of cytochrome c [107,113] during APAP-induced liver injury, there is no activation of caspases [114,115] and all morphological characteristics of necrosis including cell and organelle swelling, cell contents release and karyolysis are evident both *in vivo* and *in vitro* [116-118]. In addition, caspase inhibitors do not protect against APAP-induced liver injury [114,115,118, 119]. Recent studies have uncovered a number of molecular mediators dictating the mode of cell death after APAP overdose, which confirms that cells die by a controlled form of cell death, now termed regulated necrosis. Necroptosis, a form of regulated necrosis is typically mediated by activation of death receptors such as TNF receptor 1 (TNFR1), which ultimately lead to the assembly of a necrotic death complex (necrosome) [120], which consists of the receptor-interacting kinase 1 (RIP1), the receptor-interacting kinase 3 (RIP3), which interact through their homotypic interaction motif (RHIM) domains, as well as the pseudokinase mixed-lineage kinase domain-like protein (MLKL) [121]. The activation of these mediators such as the receptor interacting protein kinases 1 and 3 (RIPK 1 and 3), result in necrosis while inhibiting apoptosis [122], and RIP3 has been suggested to be a molecular switch between apoptosis and necrosis [123] implicating it as a unique molecular regulator dictating necrotic cell death. It has been suggested that death signals flows from RIP1 to RIP3 through their RHIM domains, resulting in recruitment of additional RIP3 molecules for signal propagation, implying that RIP3 oligomerization is the minimal functional unit that is required to drive necrosome assembly [121]. Activated RIP3 binds to MLKL, and subsequently phosphorylates MLKL [124]. Activated MLKL then traffics the necrosome to various phospholipid-rich cellular compartments, where MLKL interferes with membrane integrity, causing necrotic cell death [121].

Early induction of RIP3 levels were evident after APAP overdose [73,125], and genetic deletion of RIP3 delayed APAP-induced cell death [73], implicating the kinase in APAP-induced cell death pathways. This involvement of RIP3 in APAP-induced acute liver injury is supported by several reports showing upregulation of RIP3 protein expression in ethanol-induced liver injury. [126], non-alcoholic steatohepatitis [127] and furosemide-induced liver injury [128]. It was also recently reported that while RIP3 deletion was protective in ConA-induced autoimmune hepatitis, RIP1 inhibition exacerbated disease and accelerated animal death. In APAP-mediated liver injury however, blockade of either RIP1 or RIP3 was protective [125]. In addition, a recently developed, highly selective RIP3 inhibitor protected against APAP-induced cell death in isolated human hepatocytes in vitro and in an in vivo mouse model. [129]. In contrast to all this evidence, one group of researchers were unable to detect RIP3 expression in primary mouse hepatocytes under basal conditions or after treatment with APAP [74], a finding which could have been influenced by culture conditions [130].

It should be noted however, that the various physiological and pathophysiological processes may have slight differences in specific components of the necrosome, with inducers such as the murine cytomegalovirus (MCMV) and herpes simplex virus having other proteins with RHIM domains substituting for necrosome components[121]. Hence, requirements of specific necrosome components may be variable depending on the pathophysiology, with Toll-like receptor 3-mediated necrosis proceeding independent of RIP1 [131] and MLKL-mediated regulated necrosis proceeding independently of RIPK3 in inflammation after Con A-induced hepatitis [132]. The involvement of MLKL in APAP-induced liver injury is questionable however, since two studies found that MLKL knockout mice were not protected [74,132]. This information, coupled with the fact that APAP hepatotoxicity is not attenuated in absence of TNFR1 [133] or influenced by absence of TNF [134], leads to the conclusion that APAP-induced cell death probably does not involve classical necroptosis. However, reactive oxygen species, which are central players in the APAP-induced signaling cascade, have been shown to be involved in RIP3-induced necroptosis in the cardiomyocyte [135] and several studies have also implicated RIP1 in APAP-induced liver injury [57,73,74,136] further confirming that the mode of cell death after APAP is regulated necrosis. A recent report has identified an iron dependent non-apoptotic form of cell death termed ferroptosis [137] and studies on primary hepatocytes in culture seem to suggest that this form of cell death may play a role in APAP-induced hepatocyte injury [138]. However, the physiological significance of this form of cell death in APAP-induced liver injury in vivo needs further study. Thus, based on the current data in the literature, APAP-induced hepatocyte cell death does not represent classical apoptosis or necroptosis. While the exact sequence of events and identity of all the mediators involved in the process are still being investigated, hepatocytes after an APAP overdose die by regulated necrosis.

## 9. Role of inflammation and intercellular communication in APAP-induced liver injury

APAP-induced hepatocyte necrosis results in massive release of damage associated molecular patterns (DAMPs), which can then lead to recruitment of monocytes and neutrophils. [139]. While it is well established that inflammation is induced after APAP- induced liver injury, there has been some controversy in the literature regarding the biological role of this inflammation and whether it would be a useful therapeutic target. While a number of earlier studies suggested that inflammation played a role in hepatocyte necrosis with P2X7 receptor-mediated purinergic signaling thought to promote liver injury through the inflammasome [140,141], the identity of the cytotoxic cell type involved in immune mediated injury is not clear and the effect of P2X7 was shown to be due to inhibition of P450 isoenzymes by the inhibitor of P2X7 and not through inflammasome activation [142]. A considerable amount of data, reviewed in Woolbright et al. [139], exists that raises concerns about the role of sterile inflammation and the importance of inflammasome activation during APAP hepatotoxicity, and the majority of experimental evidence suggests that the extensive sterile inflammatory response during APAP hepatotoxicity is mainly beneficial by limiting the formation and the impact of pro-inflammatory mediators and by promoting tissue repair [143]. The role of inflammation in APAP-induced liver injury is further discussed in detail in the Woolbright article in this special issue [144].

Intercellular communication plays an important role in tissue homeostasis, and gap junctions between hepatocyte are important guardians of this process. The gap junctions are composed of connexin proteins [145] and a small molecule inhibitor of connexin 32 (2-aminoethoxy-diphenyl-borate) was suggested to be protective against APAP hepatotoxicity [146]. However, it was later shown that the 2-aminoethoxy-diphenyl-borate protects against acetaminophen hepatotoxicity by inhibiting cytochrome P450 enzymes and JNK activation [147]. Genetic deficiency of connexin32 was also found to have no effect on acetaminophen-induced cell death, inflammation or oxidative stress [148]. However, a study exploring the role of multiple connexins such as connexin26, connexin32 and connexin43 demonstrated that gap junction communication was compromised after APAP overdose, accompanied by a switch in connexin production from connexin32 and connexin26 to connexin43 [149]. Connexin43-deficient animals had aggravated liver injury after APAP overdose, with increased cell death, inflammation and oxidative stress, suggesting that hepatic connexin43-mediated signaling could protect against APAP-induced liver injury [149]. A recent study also suggests that inhibition of pannexins, a family of transmembrane channel forming proteins linking the cytosol to the extracellular environment, alleviates APAP-induced hepatotoxicity [150]. Thus, the intracellular signaling which results in hepatocyte necrosis after APAP could also influence inter-cellular communication through various gap junction proteins and further research in this area will provide additional insight into the transmission of APAP- induced signaling across the liver. Further details of the role of gap junctions in APAP hepatotoxicity are discussed in the Maes article in this special issue [151].

## 10. Translation of mechanisms of APAP-induced liver injury from mice to humans

The translation of mechanisms obtained from mouse models of APAP overdose to the human situation were only initiated recently. Exploration of intra-hepatic changes after APAP overdose in humans can only be studied using in vitro cell culture approaches, due to the lack of liver biopsies from these patients where the procedure is contraindicated. A major constraint towards these in vitro analyses was the metabolic incompetence of the commonly used liver cell line HepG2 [152]. However, recent studies in metabolically competent HepaRG cell lines [23] and primary human hepatocytes [66] have confirmed that exposure to high levels of APAP result in GSH depletion, APAP protein adduct formation in mitochondria and organelle dysfunction accompanied by oxidant stress, resulting in necrotic cell death in human cells as demonstrated earlier in mouse models. JNK activation and its translocation to the mitochondria were also evident in primary hepatocytes [66], while this was not seen in HepaRG cells [66]. Thus, many key intracellular signaling events discovered in mice are reproducible in human hepatocytes suggesting that common mechanisms are in play during progression of APAP-induced liver injury. Further confirmation of the similarity of mechanisms comes from the detection of APAP protein adducts in plasma from patients after APAP overdose implying that they generate a similar reactive metabolite as in mice [153]. Though APAP protein adduct levels could identify patients with potential APAP overdose [16,153], interpretation needs to be tempered by the fact that APAP protein adducts were detectable in circulation in humans taking therapeutic doses of acetaminophen and this persisted for over a week after dosing was stopped [154]. Also, protein adduct levels tend to peak at later time points in humans [18] when compared to mice [15], which correlates with the delayed cell injury in patients and human hepatocytes.

The central role of mitochondrial dysfunction and nuclear DNA fragmentation in APAP-induced liver injury in the mouse model is also replicated in humans as illustrated by the detection of a mitochondrial matrix enzyme, glutamate dehydrogenase, mitochondrial DNA and nuclear DNA fragments in patients with APAP overdose [155]. The importance of mitochondrial dysfunction is further confirmed by the correlation of these serum markers of mitochondrial damage with poor outcome, as defined by death or need for liver transplant to survive [156]. Confirmation of the mode of cell death in humans as being necrosis comes from the significant elevation in biomarkers of necrosis, such as full-length cytokeratin-18, HMGB1 and microRNA-122 in APAP overdose patients [157, 158] in contrast to apoptotic biomarkers such as the caspase-cleaved form of cytokeratin-18 and caspase-3 activity, which showed only a minor increase or no significant change, respectively, in these patients [155,157]. Thus, experiments in primary human hepatocytes and human HepaRG cells as well as measurement of specific circulating biomarkers from patients after APAP overdose indicate commonality of mechanisms of liver injury in mice and men. It is important to recognize that rats are generally less susceptible to APAP than mice and are not a good model for the human toxicity [25]. From an intervention standpoint, administration of antibodies against HMGB1 has been shown to be protective against APAP-induced liver injury, by enhancing liver regeneration and recovery [159]. HMGB1 neutralization also decreased bacterial translocation from the gut during APAP hepatotoxicity [160]. A recent study showed that use of a partly humanized anti-HMGB1 monoclonal antibody in mice resulted in a 50% reduction in liver injury after APAP overdose, with prolonged therapeutic efficacy when compared to NAC [161]. Interestingly, however, animals treated with the humanized antibody showed reduced regeneration [161], suggesting that mechanisms of protection were different from that seen with the rodent anti-HMGB1 treatment [159].

In addition to information of mechanisms of liver injury, prediction of the course of liver injury and severity in individual patients would be very important for APAP overdose patients from the clinical management standpoint. Measurement of liver enzymes such as ALT and AST are not very useful in this context, since they represent acute cell death and peak levels of ALT and AST are not predictive of clinical outcome [157,162]. Serum biomarkers which could predict acute liver failure and death, would hence be of immense clinical use. Towards this end, it is now evident that higher levels of cytokeratin-18, HMGB1 and glycodeoxycholic acid levels correlated with poor outcome [157,163]. At the other end of spectrum, in early presenting patients, elevations in miR-122, cytokeratin-18, HMGB1 and argininosuccinate synthetase could indicate liver injury before ALT/AST increases are measurable [162,164,165]. Profiles of circulating miRNA could also be useful in identifying APAP hepatotoxicity and in differentiating cause of cell damage [166]. Thus, key mechanistic steps in the cellular signaling cascade induced in the liver after APAP overdose first discovered in animal models have been corroborated in human hepatocytes and also indirectly by circulating biomarkers from human patients. Future studies in this area of biomarker discovery is likely to uncover additional molecules, which could identify therapeutic targets or help predict the course of liver injury after APAP overdose in the clinic [162].

In conclusion, significant advances have been made in recent years regarding cell signaling mechanisms involved in APAP-induced liver injury, with the role of mitochondrial dynamics and regulated necrosis being identified. A number of these key mechanistic events are also reproduced in humans, suggesting that cellular responses to APAP overdose are conserved between mice and men. However, significant lacunae exist in our knowledge regarding a number of steps in the process, justifying further studies on mechanisms as well as predictive biomarkers in the future.

## ABBREVIATIONS

APAP: Acetaminophen;
AMAP: 3’-hydroxyacetanilide;
NAPQI: *N*-acetyl-*p*-benzoquinone imine;
JNK: c-jun *N*-terminal kinase;
RIP3: receptor-interacting protein kinase 3;
DRP 1: dynamin related protein 1;
MPT: mitochondrial membrane permeability transition;
AIF: apoptosis inducing factor;
ALF: acute liver failure;
NAC: *N*-acetylcysteine;
PARK7: Parkinson disease protein 7;
VDAC2: voltage-dependent anion-selective channel protein 2;
SOD2: manganese superoxide dismutase;
ONOO: peroxynitrite;
iNOS: inducible nitric oxide synthase;
nNOS: neuronal nitric oxide synthase;
ASK1: apoptosis signal-regulating kinase 1;
OPA1: optic atrophy 1 protein;
Mfn1/2: mitofusin 1/2;
Fis1: mitochondrial fission 1 protein;
MOMP: mitochondrial outer membrane permeabilization;
Smac: *s*econd *m*itochondria-derived *a*ctivator of *c*aspase;
HMGB1: high mobility group box 1 protein

## References

[1] Larson AM, Polson J, Fontana RJ, Davern TJ, Lalani E, Hynan LS, Reisch JS, Schiødt FV, Ostapowicz G, Shakil AO, Lee WM (2005). Acute Liver Failure Study Group. Acetaminophen-induced acute liver failure: results of a United States multicenter, prospective study. Hepatology.

[2] Reuben A, Tillman H, Fontana RJ, Davern T, McGuire B, Stravitz RT, Durkalski V, Larson AM, Liou I, Fix O, Schilsky M, McCashland T, Hay JE, Murray N, Shaikh OS, Ganger D, Zaman A, Han SB, Chung RT, Smith A, Brown R, Crippin J, Harrison ME, Koch D, Munoz S, Reddy KR, Rossaro L, Satyanarayana R, Hassanein T, Hanje AJ, Olson J, Subramanian R, Karvellas C, Hameed B, Sherker AH, Robuck P, Lee WM (2016). Outcomes in adults with acute liver failure between 1998 and 2013: an observational cohort study. Ann Intern Med.

[3] Lancaster EM, Hiatt JR, Zarrinpar A (2015). Acetaminophen hepatotoxicity: an updated review. Arch Toxicol.

[4] Clark R, Fisher JE, Sketris IS, Johnston GM (2012). Population prevalence of high dose paracetamol in dispensed paracetamol/opioid prescription combinations: an observational study. BMC Clin Pharmacol.

[5] Maes M, Vinken M, Jaeschke H (2016). Experimental models of hepatotoxicity related to acute liver failure. Toxicol Appl Pharmacol.

[6] Jaeschke H, Xie Y, McGill MR (2014). Acetaminophen-induced liver injury: from animal models to humans. J Clin Transl Hepatol.

[7] Mitchell JR, Jollow DJ, Potter WZ, Gillette JR, Brodie BB (1973). Acetaminophen-induced hepatic necrosis. IV. Protective role of glutathione. J Pharm Exp Ther.

[8] Prescott LF, Park J, Ballantyne A, Adriaenssens P, Proudfoot AT (1977). Treatment of paracetamol (acetaminophen) poisoning with N-acetylcysteine. Lancet.

[9] Peterson RG, Rumack BH (1977). Treating acute acetaminophen poisoning with acetylcysteine. JAMA.

[10] McGill MR, Jaeschke H (2013). Metabolism and disposition of acetaminophen: recent advances in relation to hepatotoxicity and diagnosis. Pharm Res.

[11] Zaher H, Buters JT, Ward JM, Bruno MK, Lucas AM, Stern ST, Cohen SD, Gonzalez FJ (1998). Protection against acetaminophen toxicity in CYP1A2 and CYP2E1 double-null mice. Toxicol Appl Pharmacol.

[12] Nelson SD (1990). Molecular mechanisms of the hepatotoxicity caused by acetaminophen. Seminars in liver disease.

[13] Hinson JA, Monks TJ, Hong M, Highet RJ, Pohl LR (1982). 3-(glutathion-S-yl)acetaminophen: a biliary metabolite of acetaminophen. Drug Metab Dispos.

[14] Chen C, Hennig GE, Manautou JE (2003). Hepatobiliary excretion of acetaminophen glutathione conjugate and its derivatives in transport-deficient (TR-) hyperbilirubinemic rats. Drug Metab Dispos.

[15] McGill MR, Lebofsky M, Norris HR, Slawson MH, Bajt ML, Xie Y, Williams CD, Wilkins DG, Rollins DE, Jaeschke H (2013). Plasma and liver acetaminophen-protein adduct levels in mice after acetaminophen treatment: dose-response, mechanisms, and clinical implications. Toxicol Appl Pharmacol.

[16] Heard KJ, Green JL, James LP, Judge BS, Zolot L, Rhyee S, Dart RC (2011). Acetaminophen-cysteine adducts during therapeutic dosing and following overdose. BMC Gastroenterol.

[17] Ni HM, McGill MR, Chao X, Du K, Williams JA, Xie Y, Jaeschke H, Ding WX (2016). Removal of acetaminophen protein adducts by autophagy protects against acetaminophen-induced liver injury in mice. J Hepatology.

[18] Xie Y, McGill MR, Cook SF, Sharpe MR, Winefield RD, Wilkins DG, Rollins DE, Jaeschke H (2015). Time course of acetaminophen-protein adducts and acetaminophen metabolites in circulation of overdose patients and in HepaRG cells. Xenobiotica.

[19] Jaeschke H (1990). Glutathione disulfide formation and oxidant stress during acetaminophen-induced hepatotoxicity in mice in vivo: the protective effect of allopurinol. J Pharm Exp Ther.

[20] Du K, Williams CD, McGill MR, Jaeschke H (2014). Lower susceptibility of female mice to acetaminophen hepatotoxicity: Role of mitochondrial glutathione, oxidant stress and c-jun N-terminal kinase. Toxicol Appl Pharmacol.

[21] Soga T, Baran R, Suematsu M, Ueno Y, Ikeda S, Sakurakawa T, Kakazu Y, Ishikawa T, Robert M, Nishioka T, Tomita M (2006). Differential metabolomics reveals ophthalmic acid as an oxidative stress biomarker indicating hepatic glutathione consumption. J Biol Chem.

[22] Kaur G, Leslie EM, Tillman H, Lee WM, Swanlund DP, Karvellas CJ (2015). US Acute Liver Failure Study Group. Detection of ophthalmic acid in serum from acetaminophen-induced acute liver failure patients is more frequent in non-survivors. PLoS One.

[23] McGill MR, Yan HM, Ramachandran A, Murray GJ, Rollins DE, Jaeschke H (2011). HepaRG cells: a human model to study mechanisms of acetaminophen hepatotoxicity. Hepatology.

[24] Tirmenstein MA, Nelson SD (1989). Subcellular binding and effects on calcium homeostasis produced by acetaminophen and a nonhepatotoxic regioisomer, 3’-hydroxyacetanilide, in mouse liver. J Biol Chem.

[25] McGill MR, Williams CD, Xie Y, Ramachandran A, Jaeschke H (2012). Acetaminophen-induced liver injury in rats and mice: comparison of protein adducts, mitochondrial dysfunction, and oxidative stress in the mechanism of toxicity. Toxicol Appl Pharmacol.

[26] Hu J, Ramshesh VK, McGill MR, Jaeschke H, Lemasters JJ (2016). Low Dose Acetaminophen Induces Reversible Mitochondrial Dysfunction Associated with Transient c-Jun N-Terminal Kinase Activation in Mouse Liver. Toxicol Sci.

[27] Baines CP (2010). Role of the mitochondrion in programmed necrosis. Front Physiol.

[28] Myers TG, Dietz EC, Anderson NL, Khairallah EA, Cohen SD, Nelson SD (1995). A comparative study of mouse liver proteins arylated by reactive metabolites of acetaminophen and its nonhepatotoxic regioisomer, 3’-hydroxyacetanilide. Chem Res Toxicol.

[29] Matthews AM, Hinson JA, Roberts DW, Pumford NR (1997). Comparison of covalent binding of acetaminophen and the regioisomer 3’-hydroxyacetanilide to mouse liver protein. Toxicol Lett.

[30] Hadi M, Dragovic S, van Swelm R, Herpers B, van de Water B, Russel FG, Commandeur JN, Groothuis GM (2013). AMAP, the alleged non-toxic isomer of acetaminophen, is toxic in rat and human liver. Arch Toxicol.

[31] Xie Y, McGill MR, Du K, Dorko K, Kumer SC, Schmitt TM, Ding WX, Jaeschke H (2015). Mitochondrial protein adducts formation and mitochondrial dysfunction during N-acetyl-m-aminophenol (AMAP)-induced hepatotoxicity in primary human hepatocytes. Toxicol Appl Pharmacol.

[32] Qiu Y, Benet LZ, Burlingame AL (1998). Identification of the hepatic protein targets of reactive metabolites of acetaminophen in vivo in mice using two-dimensional gel electrophoresis and mass spectrometry. J Biol Chem.

[33] Andringa KK, Bajt ML, Jaeschke H, Bailey SM (2008). Mitochondrial protein thiol modifications in acetaminophen hepatotoxicity: effect on HMG-CoA synthase. Toxicol Lett.

[34] Bruderer R, Bernhardt OM, Gandhi T, Miladinovic SM, Cheng LY, Messner S, Ehrenberger T, Zanotelli V, Butscheid Y, Escher C, Vitek O, Rinner O, Reiter L (2015). Extending the limits of quantitative proteome profiling with data-independent acquisition and application to acetaminophen-treated three-dimensional liver microtissues. Mol Cell Proteomics.

[35] Jaeschke H, Knight TR, Bajt ML (2003). The role of oxidant stress and reactive nitrogen species in acetaminophen hepatotoxicity. Toxicol Lett.

[36] Yan HM, Ramachandran A, Bajt ML, Lemasters JJ, Jaeschke H (2010). The oxygen tension modulates acetaminophen-induced mitochondrial oxidant stress and cell injury in cultured hepatocytes. Toxicol Sci.

[37] Meyers LL, Beierschmitt WP, Khairallah EA, Cohen SD (1988). Acetaminophen-induced inhibition of hepatic mitochondrial respiration in mice. Toxicol Appl Pharmacol.

[38] Ramachandran A, Lebofsky M, Weinman SA, Jaeschke H (2011). The impact of partial manganese superoxide dismutase (SOD2)- deficiency on mitochondrial oxidant stress, DNA fragmentation and liver injury during acetaminophen hepatotoxicity. Toxicol Appl Pharmacol.

[39] Fujimoto K, Kumagai K, Ito K, Arakawa S, Ando Y, Oda S, Yamoto T, Manabe S (2009). Sensitivity of liver injury in heterozygous Sod2 knockout mice treated with troglitazone or acetaminophen. Toxicol Pathol.

[40] Radi R, Peluffo G, Alvarez MN, Naviliat M, Cayota A (2001). Unraveling peroxynitrite formation in biological systems. Free Radic Biol Med.

[41] Cover C, Mansouri A, Knight TR, Bajt ML, Lemasters JJ, Pessayre D, Jaeschke H (2005). Peroxynitrite-induced mitochondrial and endonuclease- mediated nuclear DNA damage in acetaminophen hepatotoxicity. J Pharmacol Exp Ther.

[42] Saito C, Lemasters JJ, Jaeschke H (2010). c-Jun N-terminal kinase modulates oxidant stress and peroxynitrite formation independent of inducible nitric oxide synthase in acetaminophen hepatotoxicity. Toxicol Appl Pharmacol.

[43] Hinson JA, Bucci TJ, Irwin LK, Michael SL, Mayeux PR (2002). Effect of inhibitors of nitric oxide synthase on acetaminophen-induced hepatotoxicity in mice. Nitric Oxide.

[44] Michael SL, Mayeux PR, Bucci TJ, Warbritton AR, Irwin LK, Pumford NR, Hinson JA (2001). Acetaminophen-induced hepatotoxicity in mice lacking inducible nitric oxide synthase activity. Nitric Oxide.

[45] Villanueva C, Giulivi C (2010). Subcellular and cellular locations of nitric oxide synthase isoforms as determinants of health and disease. Free Radic Biol Med.

[46] Banerjee S, Melnyk SB, Krager KJ, Aykin-Burns N, Letzig LG, James LP, Hinson JA (2015). The neuronal nitric oxide synthase inhibitor NANT blocks acetaminophen toxicity and protein nitration in freshly isolated hepatocytes. Free Radic Biol Med.

[47] Agarwal R, Hennings L, Rafferty TM, Letzig LG, McCullough S, James LP, MacMillan-Crow LA, Hinson JA (2012). Acetaminophen-induced hepatotoxicity and protein nitration in neuronal nitric-oxide synthase knockout mice. J Pharmocol Exp Ther.

[48] Agarwal R, MacMillan-Crow LA, Rafferty TM, Saba H, Roberts DW, Fifer EK, James LP, Hinson JA (2011). Acetaminophen-induced hepatotoxicity in mice occurs with inhibition of activity and nitration of mitochondrial manganese superoxide dismutase. J Pharmacol Exp Ther.

[49] Knight TR, Ho YS, Farhood A, Jaeschke H (2002). Peroxynitrite is a critical mediator of acetaminophen hepatotoxicity in murine livers: protection by glutathione. J Pharmocol Exp Ther.

[50] Bajt ML, Knight TR, Farhood A, Jaeschke H (2003). Scavenging peroxynitrite with glutathione promotes regeneration and enhances survival during acetaminophen-induced liver injury in mice. J Pharmocol Exp Ther.

[51] James LP, McCullough SS, Lamps LW, Hinson JA (2003). Effect of N-acetylcysteine on acetaminophen toxicity in mice: relationship to reactive nitrogen and cytokine formation. Toxicol Sci.

[52] Saito C, Zwingmann C, Jaeschke H (2010). Novel mechanisms of protection against acetaminophen hepatotoxicity in mice by glutathione and N-acetylcysteine. Hepatology.

[53] Du K, Farhood A, Jaeschke H (2017). Mitochondria-targeted antioxidant Mito-Tempo protects against acetaminophen hepatotoxicity. Arch Toxicol.

[54] Hanawa N, Shinohara M, Saberi B, Gaarde WA, Han D, Kaplowitz N (2008). Role of JNK translocation to mitochondria leading to inhibition of mitochondria bioenergetics in acetaminophen-induced liver injury. J Biol Chem.

[55] Nakagawa H, Maeda S, Hikiba Y, Ohmae T, Shibata W, Yanai A, Sakamoto K, Ogura K, Noguchi T, Karin M, Ichijo H, Omata M (2008). Deletion of apoptosis signal-regulating kinase 1 attenuates acetaminophen-induced liver injury by inhibiting c-Jun N-terminal kinase activation. Gastroenterol.

[56] Xie Y, Ramachandran A, Breckenridge DG, Liles JT, Lebofsky M, Farhood A, Jaeschke H (2015). Inhibitor of apoptosis signal-regulating kinase 1 protects against acetaminophen-induced liver injury. Toxicol Appl Pharmacol.

[57] Sharma M, Gadang V, Jaeschke A (2012). Critical role for mixed-lineage kinase 3 in acetaminophen-induced hepatotoxicity. Mol Pharmacol.

[58] Lee HS, Hwang CY, Shin SY, Kwon KS, Cho KH (2014). MLK3 is part of a feedback mechanism that regulates different cellular responses to reactive oxygen species. Sci Signal.

[59] Win S, Than TA, Han D, Petrovic LM, Kaplowitz N (2011). c-Jun N-terminal kinase (JNK)-dependent acute liver injury from acetaminophen or tumor necrosis factor (TNF) requires mitochondrial Sab protein expression in mice. J Biol Chem.

[60] Win S, Than TA, Min RW, Aghajan M, Kaplowitz N (2016). c-Jun N-terminal kinase mediates mouse liver injury through a novel Sab (SH3BP5)-dependent pathway leading to inactivation of intramitochondrial Src. Hepatology.

[61] Cubero FJ, Zoubek ME, Hu W, Peng J, Zhao G, Nevzorova YA, Al Masaoudi M, Bechmann LP, Boekschoten MV, Muller M, Preisinger C, Gassler N, Canbay AE, Luedde T, Davis RJ, Liedtke C, Trautwein C (2016). Combined Activities of JNK1 and JNK2 in Hepatocytes Protect Against Toxic Liver Injury. Gastroenterol.

[62] Bourdi M, Korrapati MC, Chakraborty M, Yee SB, Pohl LR (2008). Protective role of c-Jun N-terminal kinase 2 in acetaminophen-induced liver injury. Biochem Biophys Res Commun.

[63] Bourdi M, Davies JS, Pohl LR (2011). Mispairing C57BL/6 substrains of genetically engineered mice and wild-type controls can lead to confounding results as it did in studies of JNK2 in acetaminophen and concanavalin A liver injury. Chem Res Toxicol.

[64] Gunawan BK, Liu ZX, Han D, Hanawa N, Gaarde WA, Kaplowitz N (2006). c-Jun N-terminal kinase plays a major role in murine acetaminophen hepatotoxicity. Gastroenterol.

[65] Henderson NC, Pollock KJ, Frew J, Mackinnon AC, Flavell RA, Davis RJ, Sethi T, Simpson KJ (2007). Critical role of c-jun (NH2) terminal kinase in paracetamol-induced acute liver failure. Gut.

[66] Xie Y, McGill MR, Dorko K, Kumer SC, Schmitt TM, Forster J, Jaeschke H (2014). Mechanisms of acetaminophen-induced cell death in primary human hepatocytes. Toxicol Appl Pharmacol.

[67] Bajt ML, Farhood A, Lemasters JJ, Jaeschke H (2008). Mitochondrial bax translocation accelerates DNA fragmentation and cell necrosis in a murine model of acetaminophen hepatotoxicity. J Pharmacol Exp Ther.

[68] El-Hassan H, Anwar K, Macanas-Pirard P, Crabtree M, Chow SC, Johnson VL, Lee PC, Hinton RH, Price SC, Kass GE (2003). Involvement of mitochondria in acetaminophen-induced apoptosis and hepatic injury: roles of cytochrome c, Bax, Bid, and caspases. Toxicol Appl Pharmacol.

[69] Karch J, Molkentin JD (2015). Regulated necrotic cell death: the passive aggressive side of Bax and Bak. Circ Res.

[70] Silva Ramos E, Larsson NG, Mourier A (2016). Bioenergetic roles of mitochondrial fusion. Biochim Biophys Acta.

[71] Ishihara T, Kohno H, Ishihara N (2015). Physiological roles of mitochondrial fission in cultured cells and mouse development. Ann N Y Acad Sci.

[72] Zhao J, Lendahl U, Nister M (2013). Regulation of mitochondrial dynamics: convergences and divergences between yeast and vertebrates. Cell Mol Life Sci.

[73] Ramachandran A, McGill MR, Xie Y, Ni HM, Ding WX, Jaeschke H (2013). Receptor interacting protein kinase 3 is a critical early mediator of acetaminophen-induced hepatocyte necrosis in mice. Hepatology.

[74] Dara L, Johnson H, Suda J, Win S, Gaarde W, Han D, Kaplowitz N (2015). Receptor interacting protein kinase 1 mediates murine acetaminophen toxicity independent of the necrosome and not through necroptosis. Hepatology.

[75] Karbowski M, Lee YJ, Gaume B, Jeong SY, Frank S, Nechushtan A, Santel A, Fuller M, Smith CL, Youle RJ (2002). Spatial and temporal association of Bax with mitochondrial fission sites, Drp1, and Mfn2 during apoptosis. J Cell Biol.

[76] Wu S, Zhou F, Zhang Z, Xing D (2011). Bax is essential for Drp1-mediated mitochondrial fission but not for mitochondrial outer membrane permeabilization caused by photodynamic therapy. J Cell Physiol.

[77] Wang P, Wang P, Liu B, Zhao J, Pang Q, Agrawal SG, Jia L, Liu FT (2015). Dynamin-related protein Drp1 is required for Bax translocation to mitochondria in response to irradiation-induced apoptosis. Onco-target.

[78] Montessuit S, Somasekharan SP, Terrones O, Lucken-Ardjomande S, Herzig S, Schwarzenbacher R, Manstein DJ, Bossy-Wetzel E, Basañez G, Meda P, Martinou JC (2010). Membrane remodeling induced by the dynamin-related protein Drp1 stimulates Bax oligomerization. Cell.

[79] Cassidy-Stone A, Chipuk JE, Ingerman E, Song C, Yoo C, Kuwana T, Kurth MJ, Shaw JT, Hinshaw JE, Green DR, Nunnari J (2008). Chemical inhibition of the mitochondrial division dynamin reveals its role in Bax/Bak-dependent mitochondrial outer membrane permeabilization. Dev Cell.

[80] Ni HM, Bockus A, Boggess N, Jaeschke H, Ding WX (2012). Activation of autophagy protects against acetaminophen-induced hepatotoxicity. Hepatology.

[81] Ni HM, Williams JA, Jaeschke H, Ding WX (2013). Zonated induction of autophagy and mitochondrial spheroids limits acetaminophen-induced necrosis in the liver. Redox Biol.

[82] Williams JA, Ni HM, Haynes A, Manley S, Li Y, Jaeschke H, Ding WX (2015). Chronic deletion and acute knockdown of Parkin have differential responses to acetaminophen-induced mitophagy and liver in-jury in mice. J Biol Chem.

[83] Ding WX, Guo F, Ni HM, Bockus A, Manley S, Stolz DB, Eskelinen EL, Jaeschke H, Yin XM (2012). Parkin and mitofusins reciprocally regulate mitophagy and mitochondrial spheroid formation. J Biol Chem.

[84] Ni HM, Boggess N, McGill MR, Lebofsky M, Borude P, Apte U, Jaeschke H, Ding WX (2012). Liver-specific loss of Atg5 causes persistent activation of Nrf2 and protects against acetaminophen-induced liver injury. Toxicol Sci.

[85] Ramachandran A, Lebofsky M, Yan HM, Weinman SA, Jaeschke H (2015). Hepatitis C virus structural proteins can exacerbate or ameliorate acetaminophen-induced liver injury in mice. Arch Toxicol.

[86] Nagy G, Kardon T, Wunderlich L, Szarka A, Kiss A, Schaff Z, Bánhegyi G, Mandl J (2007). Acetaminophen induces ER dependent signaling in mouse liver. Arch Biochem Biophys.

[87] Uzi D, Barda L, Scaiewicz V, Mills M, Mueller T, Gonzalez-Rodriguez A, Valverde AM, Iwawaki T, Nahmias Y, Xavier R, Chung RT, Tirosh B, Shibolet O (2013). CHOP is a critical regulator of acetaminophen-induced hepatotoxicity. J Hepatol.

[88] Foufelle F, Fromenty B (2016). Role of endoplasmic reticulum stress in drug-induced toxicity. Pharmacol Res Perspect.

[89] Zoratti M, Szabo I (1995). The mitochondrial permeability transition. Biochim. Biophys Acta.

[90] Karch J, Kwong JQ, Burr AR, Sargent MA, Elrod JW, Peixoto PM, Martinez-Caballero S, Osinska H, Cheng EH, Robbins J, Kinnally KW, Molkentin JD (2013). Bax and Bak function as the outer membrane component of the mitochondrial permeability pore in regulating necrotic cell death in mice. eLife.

[91] Karch J, Molkentin JD (2014). Identifying the components of the elusive mitochondrial permeability transition pore. Proc Natl Acad Sci U S A.

[92] Baines CP, Kaiser RA, Purcell NH, Blair NS, Osinska H, Hambleton MA, Brunskill EW, Sayen MR, Gottlieb RA, Dorn GW, Robbins J, Molkentin JD (2005). Loss of cyclophilin D reveals a critical role for mitochondrial permeability transition in cell death. Nature.

[93] Alavian KN, Beutner G, Lazrove E, Sacchetti S, Park HA, Licznerski P, Li H, Nabili P, Hockensmith K, Graham M, Porter GA, Jonas EA (2014). An uncoupling channel within the c-subunit ring of the F1FO ATP synthase is the mitochondrial permeability transition pore. Proc Natl Acad Sci U S A.

[94] Kon K, Kim JS, Jaeschke H, Lemasters JJ (2004). Mitochondrial permeability transition in acetaminophen-induced necrosis and apoptosis of cultured mouse hepatocytes. Hepatology.

[95] Masubuchi Y, Suda C, Horie T (2005). Involvement of mitochondrial permeability transition in acetaminophen-induced liver injury in mice. J Hepatol.

[96] Ramachandran A, Lebofsky M, Baines CP, Lemasters JJ, Jaeschke H (2011). Cyclophilin D deficiency protects against acetaminophen-induced oxidant stress and liver injury. Free Radic Res.

[97] Reid AB, Kurten RC, McCullough SS, Brock RW, Hinson JA (2005). Mechanisms of acetaminophen-induced hepatotoxicity: role of oxidative stress and mitochondrial permeability transition in freshly isolated mouse hepatocytes. J Pharmacol Exp Therap.

[98] LoGuidice A, Boelsterli UA (2011). Acetaminophen overdose-induced liver injury in mice is mediated by peroxynitrite independently of the cyclophilin D-regulated permeability transition. Hepatology.

[99] Brenner C, Moulin M (2012). Physiological roles of the permeability transition pore. Circ Res.

[100] Kwong JQ, Molkentin JD (2015). Physiological and pathological roles of the mitochondrial permeability transition pore in the heart. Cell Metab.

[101] Mnatsakanyan N, Beutner G, Porter GA, Alavian KN, Jonas EA Physiological roles of the mitochondrial permeability transition pore. J Bioenerg Biomembr 2017; in press..

[102] Kon K, Kim JS, Uchiyama A, Jaeschke H, Lemasters JJ (2010). Lysosomal iron mobilization and induction of the mitochondrial permeability transition in acetaminophen-induced toxicity to mouse hepatocytes. Toxicol Sci.

[103] Hu J, Kholmukhamedov A, Lindsey CC, Beeson CC, Jaeschke H, Lemasters JJ (2016). Translocation of iron from lysosomes to mitochondria during acetaminophen-induced hepatocellular injury: Protection by starch-desferal and minocycline. Free Radic Biol Med.

[104] Tait SW, Green DR (2010). Mitochondria and cell death: outer membrane permeabilization and beyond. Nat Rev Mol Cell Biol.

[105] Munoz-Pinedo C, Guio-Carrion A, Goldstein JC, Fitzgerald P, Newmeyer DD, Green DR (2006). Different mitochondrial intermembrane space proteins are released during apoptosis in a manner that is coordinately initiated but can vary in duration. Proc Natl Acad Sci U S A.

[106] Arnoult D, Gaume B, Karbowski M, Sharpe JC, Cecconi F, Youle RJ (2003). Mitochondrial release of AIF and EndoG requires caspase activation downstream of Bax/Bak-mediated permeabilization. EMBO J.

[107] Bajt ML, Cover C, Lemasters JJ, Jaeschke H (2006). Nuclear translocation of endonuclease G and apoptosis-inducing factor during acetaminophen-induced liver cell injury. Toxicol Sci.

[108] Norberg E, Orrenius S, Zhivotovsky B (2010). Mitochondrial regulation of cell death: processing of apoptosis-inducing factor (AIF). Biochem Biophys Res Commun.

[109] Widlak P, Garrard WT (2005). Discovery, regulation, and action of the major apoptotic nucleases DFF40/CAD and endonuclease G. J Cell Biochem.

[110] Jahr S, Hentze H, Englisch S, Hardt D, Fackelmayer FO, Hesch RD, Knippers R (2001). DNA fragments in the blood plasma of cancer patients: quantitations and evidence for their origin from apoptotic and necrotic cells. Cancer Res.

[111] Boujrad H, Gubkina O, Robert N, Krantic S, Susin SA (2007). AIF- mediated programmed necrosis: a highly regulated way to die. Cell Cycle.

[112] Bajt ML, Ramachandran A, Yan HM, Lebofsky M, Farhood A, Lemasters JJ, Jaeschke H (2011). Apoptosis-inducing factor modulates mitochondrial oxidant stress in acetaminophen hepatotoxicity. Toxicol Sci.

[113] Knight TR, Jaeschke H (2002). Acetaminophen-induced inhibition of Fas receptor-mediated liver cell apoptosis: mitochondrial dysfunction versus glutathione depletion. Toxicol Appl Pharmacol.

[114] Lawson JA, Fisher MA, Simmons CA, Farhood A, Jaeschke H (1999). Inhibition of Fas receptor (CD95)-induced hepatic caspase activation and apoptosis by acetaminophen in mice. Toxicol Appl Pharmacol.

[115] Jaeschke H, Cover C, Bajt ML (2006). Role of caspases in acetaminophen-induced liver injury. Life Sci.

[116] Jacob M, Mannherz HG, Napirei M (2007). Chromatin breakdown by deoxyribonuclease1 promotes acetaminophen-induced liver necrosis: an ultrastructural and histochemical study on male CD-1 mice. Histochem Cell Biol.

[117] Jaeschke H, Gujral JS, Bajt ML (2004). Apoptosis and necrosis in liver disease. Liver Int.

[118] Gujral JS, Knight TR, Farhood A, Bajt ML, Jaeschke H (2002). Mode of cell death after acetaminophen overdose in mice: apoptosis or oncotic necrosis?. Toxicol Sci.

[119] Williams CD, Farhood A, Jaeschke H (2010). Role of caspase-1 and interleukin-1beta in acetaminophen-induced hepatic inflammation and liver injury. Toxicol Appl Pharmacol.

[120] Vandenabeele P, Galluzzi L, Vanden Berghe T, Kroemer G (2010). Molecular mechanisms of necroptosis: an ordered cellular explosion. Nat Rev Mol Cell Biol.

[121] Zhang J, Yang Y, He W, Sun L (2016). Necrosome core machinery: MLKL. Cell Mol Life Sci.

[122] Vandenabeele P, Declercq W, Van Herreweghe F, Vanden Berghe T (2010). The role of the kinases RIP1 and RIP3 in TNF- induced necrosis. Sci Signal.

[123] Zhang DW, Shao J, Lin J, Zhang N, Lu BJ, Lin SC, Dong MQ, Han J (2009). RIP3, an energy metabolism regulator that switches TNF-induced cell death from apoptosis to necrosis. Science.

[124] Sun L, Wang H, Wang Z, He S, Chen S, Liao D, Wang L, Yan J, Liu W, Lei X, Wang X (2012). Mixed lineage kinase domain-like protein mediates necrosis signaling downstream of RIP3 kinase. Cell.

[125] Deutsch M, Graffeo CS, Rokosh R, Pansari M, Ochi A, Levie EM, Van Heerden E1, Tippens DM1, Greco S1, Barilla R1, Tomkötter L, Zambirinis CP, Avanzi N, Gulati R, Pachter HL, Torres-Hernandez A, Eisenthal A, Daley D, Miller G (2015). Divergent effects of RIP1 or RIP3 blockade in murine models of acute liver injury. Cell Death Dis.

[126] Roychowdhury S, McMullen MR, Pisano SG, Liu X, Nagy LE (2013). Absence of receptor interacting protein kinase 3 prevents ethanol-induced liver injury. Hepatology.

[127] Gautheron J, Vucur M, Reisinger F, Cardenas DV, Roderburg C, Koppe C, Kreggenwinkel K, Schneider AT, Bartneck M, Neumann UP, Canbay A, Reeves HL, Luedde M, Tacke F, Trautwein C, Heikenwalder M, Luedde T (2014). A positive feedback loop between RIP3 and JNK controls non-alcoholic steatohepatitis. EMBO Mol Med.

[128] McGill MR, Du K, Xie Y, Bajt ML, Ding WX, Jaeschke H (2015). The role of the c-Jun N-terminal kinases 1/2 and receptor-interacting protein kinase 3 in furosemide-induced liver injury. Xenobiotica.

[129] Li JX, Feng JM, Wang Y, Li XH, Chen XX, Su Y, Shen YY, Chen Y, Xiong B, Yang CH, Ding J, Miao ZH (2014). The B-Raf(V600E) inhibitor dabrafenib selectively inhibits RIP3 and alleviates acetaminophen-induced liver injury. Cell Death Dis.

[130] Yang X, Chao X, Wang ZT, Ding WX (2016). The end of RIPK1-RIPK3-MLKL-mediated necroptosis in acetaminophen-induced hepatotoxicity?. Hepatology.

[131] Kaiser WJ, Sridharan H, Huang C, Mandal P, Upton JW, Gough PJ, Sehon CA, Marquis RW, Bertin J, Mocarski ES (2013). Toll-like receptor 3-mediated necrosis via TRIF, RIP3, and MLKL. J Biol Chem.

[132] Gunther C, He GW, Kremer AE, Murphy JM, Petrie EJ, Amann K, Vandenabeele P, Linkermann A, Poremba C, Schleicher U, Dewitz C, Krautwald S, Neurath MF, Becker C, Wirtz S (2016). The pseudokinase MLKL mediates programmed hepatocellular necrosis independently of RIPK3 during hepatitis. J Clin Invest.

[133] Gardner CR, Laskin JD, Dambach DM, Chiu H, Durham SK, Zhou P, Bruno M, Gerecke DR, Gordon MK, Laskin DL (2003). Exaggerated hepatotoxicity of acetaminophen in mice lacking tumor necrosis factor receptor-1. Potential role of inflammatory mediators. Toxicol Appl Pharmacol.

[134] Boess F, Bopst M, Althaus R, Polsky S, Cohen SD, Eugster HP, Boelsterli UA (1998). Acetaminophen hepatotoxicity in tumor necrosis factor/lymphotoxin-alpha gene knockout mice. Hepatology.

[135] Zhang T, Zhang Y, Cui M, Jin L, Wang Y, Lv F, Liu Y, Zheng W, Shang H, Zhang J, Zhang M, Wu H, Guo J, Zhang X, Hu X, Cao CM, Xiao RP (2016). CaMKII is a RIP3 substrate mediating ischemia- and oxidative stress-induced myocardial necroptosis. Nat Med.

[136] Zhang YF, He W, Zhang C, Liu XJ, Lu Y, Wang H, Zhang ZH, Chen X, Xu DX (2014). Role of receptor interacting protein (RIP)1 on apoptosis-inducing factor-mediated necroptosis during acetaminophen-evoked acute liver failure in mice. Toxicol Lett.

[137] Dixon SJ, Lemberg KM, Lamprecht MR, Skouta R, Zaitsev EM, Gleason CE, Patel DN, Bauer AJ, Cantley AM, Yang WS, Morrison B, Stockwell BR (2012). Ferroptosis: an iron-dependent form of nonapoptotic cell death. Cell.

[138] Lorincz T, Jemnitz K, Kardon T, Mandl J, Szarka A (2015). Ferroptosis is involved in acetaminophen induced cell death. Pathol Oncol Res.

[139] Woolbright BL, Jaeschke H Role of the inflammasome in acetaminophen-induced liver injury and acute liver failure. J Hepatol 2017, in press..

[140] Imaeda AB, Watanabe A, Sohail MA, Mahmood S, Mohamadnejad M, Sutterwala FS, Flavell RA, Mehal WZ (2009). Acetaminophen-induced hepatotoxicity in mice is dependent on Tlr9 and the Nalp3 inflammasome. J Clin Invest.

[141] Hoque R, Sohail MA, Salhanick S, Malik AF, Ghani A, Robson SC, Mehal WZ (2012). P2X7 receptor-mediated purinergic signaling promotes liver injury in acetaminophen hepatotoxicity in mice. Am J Physiol Gastrointest Liver Physiol.

[142] Xie Y, Williams CD, McGill MR, Lebofsky M, Ramachandran A, Jaeschke H (2013). Purinergic receptor antagonist A438079 protects against acetaminophen-induced liver injury by inhibiting p450 isoenzymes, not by inflammasome activation. Toxicol Sci.

[143] Jaeschke H, Williams CD, Ramachandran A, Bajt ML (2012). Acetaminophen hepatotoxicity and repair: the role of sterile inflammation and innate immunity. Liver Int.

[144] Woolbright BL, Jaeschke H The impact of sterile inflammation in Acute Liver Injury. J Clin Transl Res 2017; in press..

[145] Vinken M (2012). Gap junctions and non-neoplastic liver disease. J Hepatol.

[146] Patel SJ, Milwid JM, King KR, Bohr S, Iracheta-Vellve A, Li M, Vitalo A, Parekkadan B, Jindal R, Yarmush ML (2012). Gap junction inhibition prevents drug-induced liver toxicity and fulminant hepatic failure. Nat Biotechnol.

[147] Du K, Williams CD, McGill MR, Xie Y, Farhood A, Vinken M, Jaeschke H (2013). The gap junction inhibitor 2-aminoethoxy-diphenyl-borate protects against acetaminophen hepatotoxicity by inhibiting cytochrome P450 enzymes and c-jun N-terminal kinase activation. Toxicol Appl Pharmacol.

[148] Maes M, McGill MR, da Silva TC, Lebofsky M, Maria Monteiro de Araujo C, Tiburcio T, Veloso Alves Pereira I, Willebrords J, Crespo Yanguas S, Farhood A, Zaidan Dagli ML, Jaeschke H, Cogliati B, Vinken M (2016). Connexin32: a mediator of acetaminophen-induced liver injury?. Toxicol Mech Methods.

[149] Maes M, McGill MR, da Silva TC, Abels C, Lebofsky M, Maria Monteiro de Araujo C, Tiburcio T, Veloso Alves Pereira I, Willebrords J, Crespo Yanguas S, Farhood A, Beschin A, Van Ginderachter JA, Zaidan Dagli ML, Jaeschke H, Cogliati B, Vinken M (2016). Involvement of connexin43 in acetaminophen-induced liver injury. Biochim Biophys Acta.

[150] Maes M, McGill MR, da Silva TC, Abels C, Lebofsky M, Weemhoff JL, Tiburcio T, Veloso Alves Pereira I, Willebrords J, Crespo Yanguas S, Farhood A, Beschin A, Van Ginderachter JA, Penuela S, Jaeschke H, Cogliati B, Vinken M Inhibition of pannexin1 channels alleviates acetaminophen-induced hepatotoxicity. Arch Toxicol 2017; in press.

[151] Maes M, Vinken M Connexin-based signaling and drug-induced hepatotoxicity. J Clin Transl Res 2017; in press.

[152] Rodriguez-Antona C, Donato MT, Boobis A, Edwards RJ, Watts PS, Castell JV, Gómez-Lechón MJ (2002). Cytochrome P450 expression in human hepatocytes and hepatoma cell lines: molecular mechanisms that determine lower expression in cultured cells. Xenobiotica.

[153] Davern TJ, James LP, Hinson JA, Polson J, Larson AM, Fontana RJ, Lalani E, Munoz S, Shakil AO, Lee WM (2006). Acute Liver Failure Study Group. Measurement of serum acetaminophen-protein adducts in patients with acute liver failure. Gastroenterol.

[154] Heard K, Green JL, Anderson V, Bucher-Bartelson B, Dart RC (2016). Paracetamol (acetaminophen) protein adduct concentrations during therapeutic dosing. Br J Clin Pharmacol.

[155] McGill MR, Sharpe MR, Williams CD, Taha M, Curry SC, Jaeschke H (2012). The mechanism underlying acetaminophen-induced hepatotoxicity in humans and mice involves mitochondrial damage and nuclear DNA fragmentation. J Clin Invest.

[156] McGill MR, Staggs VS, Sharpe MR, Lee WM, Jaeschke H (2014). Acute Liver Failure Study Group. Serum mitochondrial biomarkers and damage-associated molecular patterns are higher in acetaminophen overdose patients with poor outcome. Hepatology.

[157] Antoine DJ, Jenkins RE, Dear JW, Williams DP, McGill MR, Sharpe MR, Craig DG, Simpson KJ, Jaeschke H, Park BK (2012). Molecular forms of HMGB1 and keratin-18 as mechanistic biomarkers for mode of cell death and prognosis during clinical acetaminophen hepatotoxicity. J Hepatol.

[158] Starkey Lewis PJ, Dear J, Platt V, Simpson KJ, Craig DG, Antoine DJ, French NS, Dhaun N, Webb DJ, Costello EM, Neoptolemos JP, Moggs J, Goldring CE, Park BK (2011). Circulating microRNAs as potential markers of human drug-induced liver injury. Hepatology.

[159] Yang R, Zhang S, Cotoia A, Oksala N, Zhu S, Tenhunen J (2012). High mobility group B1 impairs hepatocyte regeneration in acetaminophen hepatotoxicity. BMC Gastroenterol.

[160] Yang R, Zou X, Tenhunen J, Zhu S, Kajander H, Koskinen ML, Tonnessen TI (2014). HMGB1 neutralization is associated with bacterial translocation during acetaminophen hepatotoxicity. BMC Gastroenterol.

[161] Lundback P, Lea JD, Sowinska A, Ottosson L, Furst CM, Steen J, Aulin C, Clarke JI, Kipar A, Klevenvall L, Yang H, Palmblad K, Park BK, Tracey KJ, Blom AM, Andersson U, Antoine DJ, Erlandsson Harris H (2016). A novel high mobility group box 1 neutralizing chimeric antibody attenuates drug-induced liver injury and postinjury inflammation in mice. Hepatology.

[162] McGill MR, Jaeschke H (2014). Mechanistic biomarkers in acetaminophen-induced hepatotoxicity and acute liver failure: from preclinical models to patients. Expert Opin Drug Metab Toxicol.

[163] Woolbright BL, McGill MR, Staggs VS, Winefield RD, Gholami P, Olyaee M, Sharpe MR, Curry SC, Lee WM, Jaeschke H (2014). Acute Liver Failure Study Group. Glycodeoxycholic acid levels as prognostic biomarker in acetaminophen-induced acute liver failure patients. Toxicol Sci.

[164] Antoine DJ, Dear JW, Lewis PS, Platt V, Coyle J, Masson M, Thanacoody RH, Gray AJ, Webb DJ, Moggs JG, Bateman DN, Goldring CE, Park BK (2013). Mechanistic biomarkers provide early and sensitive detection of acetaminophen-induced acute liver injury at first presentation to hospital. Hepatology.

[165] McGill MR, Cao M, Svetlov A, Sharpe MR, Williams CD, Curry SC, Farhood A, Jaeschke H, Svetlov SI (2014). Argininosuccinate synthetase as a plasma biomarker of liver injury after acetaminophen overdose in rodents and humans. Biomarkers.

[166] Ward J, Kanchagar C, Veksler-Lublinsky I, Lee RC, McGill MR, Jaeschke H, Curry SC, Ambros VR (2014). Circulating microRNA profiles in human patients with acetaminophen hepatotoxicity or ischemic hepatitis. Proc Natl Acad Sci U S A.

[167] Ma DKG, Stolte C, Krycer JR, James DE, O’Donoghue SI (2015). SnapShot: Insulin/IGF1 Signaling. Cell.

